# Protective Effect of *Flos Lonicerae* against Experimental Gastric Ulcers in Rats: Mechanisms of Antioxidant and Anti-Inflammatory Action

**DOI:** 10.1155/2014/596920

**Published:** 2014-12-24

**Authors:** Jung-Woo Kang, Nari Yun, Hae-Jung Han, Jeom-Yong Kim, Joo-Young Kim, Sun-Mee Lee

**Affiliations:** ^1^School of Pharmacy, Sungkyunkwan University, Seobu-ro 2066, Jangan-gu, Suwon, Gyeonggi-do 440-746, Republic of Korea; ^2^Nonclinical Team, Green Cross Corp., 107 Inyeon-ro 30 Beon-gil, Giheung-gu, Yongin, Gyeonggi-do 446-799, Republic of Korea; ^3^Green Cross Health Science Co., Ltd., 474 Dunchon-daero, Jungwon-gu, Seongnam, Gyeonggi-do 462-725, Republic of Korea

## Abstract

*Flos Lonicerae* is one of the oldest and most commonly prescribed herbs in Eastern traditional medicine to treat various inflammatory diseases. In the present study, we investigated the effects of ethyl acetate fraction of *Flos Lonicerae* (GC-7101) on experimental gastric ulcer models and its mechanisms of action in gastric ulcer healing. The pharmacological activity of GC-7101 was investigated in rats on HCl/EtOH, indomethacin, water immersion restraint stress induced acute gastric ulcer, and acetic-acid-induced subchronic gastric ulcer. To determine its gastroprotective mechanisms, gastric wall mucus secretion, mucosal PGE_2_, mucosal NO content, nuclear translocation of NF-*κ*B, mRNA expression of inflammatory cytokines, lipid peroxidation and glutathione content, and superoxide dismutase and catalase activities were measured. GC-7101 significantly attenuated development of acute gastric ulcer and accelerated the healing of acetic-acid-induced subchronic gastric ulcer. In HCl/EtOH-induced gastric ulcer, GC-7101 markedly enhanced gastric wall mucus content which was accompanied by increased mucosal PGE_2_ and NO production. Furthermore, treatment of GC-7101 exhibited anti-inflammatory and antioxidant activities as evidenced by decreased myeloperoxidase activity, NF-*κ*B translocation, inflammatory cytokines mRNA expression, and lipid peroxidation and increased glutathione content and superoxide dismutase and catalase activities. These results demonstrated that GC-7101 possesses strong antiulcerogenic effect by modulating oxidative stress and proinflammatory mediators.

## 1. Introduction

Gastric ulcers are considered a modern epidemic and reportedly affect at least 10% of the world population [1]. Gastric ulcers develop when the actions of aggressive and defensive factors in the gastric mucosa are resolved in favor of aggression. Thus, acid suppressants have been the gold standard treatment for gastric ulcers, with considerable rates of healing and symptom relief [2]; however, concerns are growing about their side effects including anacidity, weakness of the natural protective properties of gastric mucosa, and disease recurrence [3]. In this context, many studies have emphasized the importance of the gastric defense system in overcoming the limitations of acid suppressants. Several gastroprotectants not only show strong clinical efficacy but also result in significantly reduced recurrence after complete resolution of gastric ulcers [4].

The gastric mucosal defense involves secretion of luminal factors such as mucus and bicarbonate, epithelial barriers, and continuous blood flow through mucosal microvessels. These factors are modulated by secondary components of the defense system including prostaglandins (PGs) and nitric oxide (NO) [5]. PGs and NO stimulate mucus and biocarbonate synthesis and preserve gastric microcirculation [6]. Oxidative stress has been shown to disrupt natural defense system by depleting the adherent mucus layer. It not only directly increases sensitivity to mechanical forces by producing hydroxyl radicals but also indirectly aggravates inflammatory response by activating redox-sensitive transcription factors [7]. In addition, loss of the gastric mucosal defense system can enhance the inflammatory response during the development of gastric ulcers. Indeed, patients with gastric ulcers showed increased translocation of NF-*κ*B and serum release of proinflammatory cytokines and inhibition of exacerbated inflammation resulted in better outcomes [8].

*Flos Lonicerae*, also known as* Japanese honeysuckle*,* Jin Yin Hua*, or* Ren Dong*, is one of the oldest and most commonly prescribed herbs in Eastern traditional medicine [9]. It is prescribed commonly in China, Japan, and Korea for a wide array of diseases including fever from the cold, febrile disease, dysentery, carbuncles, and virulent swelling [10].* Flos Lonicerae* possesses antipyretic, antibiotic, antioxidant, and anti-inflammatory activities. Several studies reported the immunomodulatory properties of* Flos Lonicerae in vivo* and* in vitro*; an herbal formula containing* Flos Lonicerae* inhibited proinflammatory mediators such as TNF-*α* in lipopolysaccharide-stimulated RAW 264.7 macrophages [11]. Extract* of Flos Lonicerae* also protected mice against endotoxin-induced septic injury and its active composition, chlorogenic acid, was suggested to exert the pharmacological action [12]. While extracts in water, methanol, 70% ethanol, and ethyl acetate all have effective antioxidant properties, previous report demonstrated that ethyl acetate extracts exhibited the scavenging activity against total ROS, hydroxyl radicals, and peroxynitrite production [13]. Furthermore,* Flos Lonicerae* was shown to protect the esophagus against reflux esophagitis [10]. Despite its multitherapeutic potential, the effects of* Flos Lonicerae* on a gastric ulcer model have not yet been reported.

Therefore, the aim of this study was to investigate the effects of* Flos Lonicerae* on* in vivo* experimental gastric ulcer models and its mechanisms of action in gastric ulcer healing.

## 2. Materials and Methods

### 2.1. Preparation of GC-7101

GC-7101 is the standardized extract of the bud of* Lonicera japonica* Thunberg (Caprifoliaceae). This herb was supplied from China and authenticated by Medicinal Plant Resources Bank (MPRB), Gachon University (Sungnam, Korea), according to the guideline of herbal species discrimination by the DNA analysis, issued by Ministry of Food and Drug Safety. Dried buds of* Lonicera japonica* (500 g) were extracted with 80% EtOH at 60°C, and the EtOH extract was partitioned with ethyl acetate and filtered. After filtration, ethyl acetate extract was evaporated under reduced pressure and dried to yield dry extract (12.5 g, named GC-7101). The standard method evaluating the quality of GC-7101 has been established using quantitative HPLC. The contents of the 3,5-di-*O*-caffeoylquinic acid in GC-7101 is over 12%. Throughout the experiments, GC-7101 did not affect basal stomach function and the results from the vehicle-treated control rats and GC-7101-treated control rats were not different in any parameters.

### 2.2. Animal Treatment

Male Sprague-Dawley rats weighing 180–200 g (Orient Bio, Inc., Gapyeong, Korea) were housed in standardized conditions at 23 ± 1°C under a controlled 12 h light/dark cycle and maintained on standard rodent chow and tap water* ad libitum*. All animal protocols were approved by the Animal Care Committee of Sungkyunkwan University (SUSP-11-54) and were performed in accordance with the guidelines of the National Institutes of Health. Rats were euthanized by overdose of ketamine (55 mg/kg, i.p.)/xylazine (7 mg/kg, i.p.) and stomach tissues were removed.

### 2.3. Antiulcerogenic Activities

#### 2.3.1. HCl/EtOH-Induced Gastric Ulcer

Gastric ulcers were induced by HCl/EtOH treatment according to previous reports [14]. After 48 h of fasting, rats (*n* = 8 per group) were orally administered GC-7101 (25, 50, and 100 mg/kg), vehicle (5% polyvinyl pyrrolidone, PVP, BASF Corp., CA, USA) as a control, or ranitidine (30 mg/kg), or rebamipide (30 mg/kg) as positive controls, 1 h before receiving 1 mL of 150 mM HCl in 60% EtOH (p.o.). At 1 h after induction of gastric lesions, rats were sacrificed and their stomachs were immediately removed and opened along the greater curvatures. The sum of the lesion area (mm^2^) per stomach was used as a lesion index and the ulcer inhibition rate (%) was calculated as follows:

 inhibition (%) = (1 − lesion index of test animal/lesion index of vehicle-treated HCl/EtOH animal) × 100.

#### 2.3.2. Indomethacin-Induced Gastric Ulcer

Gastric ulcers were induced by indomethacin treatment according to previous reports [15]. After 48 h of fasting, rats in groups of eight were treated as above. At 1 h after treatment, all animals received 40 mg/kg of indomethacin dissolved in 5% NaHCO_3_ (p.o.) to induce acute gastric lesions. Animals were sacrificed 6 h after induction of gastric lesions and their stomachs were removed as described above. The sum of the lesion lines (mm) per stomach was used as a lesion index and the ulcer inhibition rate (%) was calculated as described above.

#### 2.3.3. Water Immersion Restraint Stress Induced Gastric Ulcer

Rats were fasted for 24 h before experiments but given free access to tap water. Fasted rats (*n* = 8 per group) were treated as above. At 30 min after treatment, animals were placed in stress cages and immersed to the xiphoid process in water at 21 ± 1°C, as described previously [16]. Gastric lesions were observed after 6 h of water immersion restraint stress (WIRS) and the lesion index (mm) and ulcer inhibition rate (%) were calculated as described above.

#### 2.3.4. Acetic-Acid-Induced Subchronic Gastric Ulcer

Gastric ulcers were induced by acetic acid treatment according to the method described by Okabe and Pfeiffer [17], with slight modifications. On the day of experiments, 24 h fasted rats were anesthetized and laparotomy was performed. Gastric ulcers were induced by injection of 50 *μ*L of 30% acetic acid in the submucosal layer of the stomach and the abdominal wall was sutured. One day after surgery, rats (*n* = 10 per group) were orally treated as above, with drugs administered for eight consecutive days at 10:00 in the morning. On the last day of treatment, the animals were fasted for 24 h and were sacrificed. Their stomachs were immediately removed and opened along the greater curvatures, and the sum of the area of lesions (mm^2^) per stomach was used as a lesion index and the ulcer inhibition rate (%) was calculated as described above.

### 2.4. Gastric Wall Mucus Content

Gastric wall mucus content was determined as described by Kitagawa et al. [18] for an HCl/EtOH-induced gastric ulcer model. Briefly, removed stomachs were weighed and immersed in 0.1% (w/v) Alcian blue (Sigma Chemical, St. Louis, MO, USA) for 2 h. Excessive dye was removed by two successive rinses (15 min each) in 0.25 M sucrose solution. Mucus-bound dye was extracted with 10 mL of 0.5 M MgCl_2_ for 2 h with intermittent shaking every 30 min. Blue extract (800 *μ*L) was shaken vigorously with an equal volume of diethyl ether and centrifuged at 500 g for 5 min. The optical density of the supernatant was measured using a spectrophotometer at a wavelength of 580 nm. The quantity of Alcian blue extract per gram of wet stomach was calculated from a standard curve.

### 2.5. Biochemical Analysis

GC-7101 at 50 mg/kg was selected as the optimal effective dose for evaluating the molecular mechanisms of GC-7101 against acute gastric lesions with HCl/EtOH treatment.

#### 2.5.1. Lipid Peroxidation and Glutathione Content

The steady-state level of malondialdehyde (MDA), the end-product of lipid peroxidation, was analyzed in stomach tissue homogenates by spectrophotometric measurement of the level of thiobarbituric acid-reactive substances at 535 nm according to the method described by Buege and Aust [19] using 1,1,3,3,-tetraethoxypropane as the standard. Total glutathione in the stomach tissue homogenate was determined spectrophotometrically at 412 nm using yeast glutathione reductase, 5,5′-dithio-bis(2-nitrobenzoic acid), and NADPH according to the method reported by Tietze [20]. The oxidized glutathione (GSSG) level was measured using the same method in the presence of 2-vinylpyridine, and the reduced glutathione (GSH) level was determined from the difference between the total glutathione and GSSG levels.

#### 2.5.2. Catalase Activity

Catalase (CAT) activity was measured as follows: 150 *μ*L of stomach tissue homogenate in phosphate buffer (50 mM, pH 7.0) was added to 75 *μ*L potassium phosphate buffer containing 20 mM H_2_O_2_. CAT activity was determined spectrophotometrically at 240 nm and defined as the amount of enzyme required to decompose 1 *μ*mol of H_2_O_2_ per minute at 25°C and pH 7.0. Results were expressed as micromoles per minute per mg protein (*μ*mol/min/mg protein).

#### 2.5.3. Superoxide Dismutase Activity

Superoxide dismutase (SOD) activity was measured based on the ability of the enzyme to inhibit the process of pyrogallol autoxidation. Briefly, stomach tissues were homogenized in 50 mmol/L phosphate buffer (pH 7.8). The homogenate was centrifuged at 1600 g for 15 min. To various concentrations of tissue supernatants, 20 *μ*L of 10 mmol/L pyrogallol solution was added and the rate of autoxidation was measured spectrophotometrically at 540 nm. SOD activity was expressed as units of SOD/g protein (1.0 U was defined as the amount of enzyme that caused 50% inhibition of pyrogallol autoxidation).

#### 2.5.4. Enzyme-Linked Immunosorbent Assay for PGE_2_

A commercial PGE_2_ enzyme-linked immunosorbent assay (ELISA) kit (Assay Designs, Ann Arbor, MI, USA) was used for quantification of the gastric mucosal PGE_2_ concentration.

#### 2.5.5. Measurement of Gastric Mucosal NO Content

A commercial nitrate/nitrite colorimetric assay kit (Cayman Chemical, Ann Arbor, MI, USA) was used for quantification of the gastric mucosal NO content.

#### 2.5.6. Measurement of Gastric MPO Activity

Myeloperoxidase (MPO) activity in stomach tissue was measured using commercially available MPO colorimetric activity assay kit (BioVision, Milpitas, CA, USA) according to the manufacturer's protocol.

#### 2.5.7. Protein Extraction of Whole Stomach Tissue

Isolated stomach tissue was homogenized in PRO-PREP Protein Extraction Solution (iNtRON Biotechnology Inc., Seongnam, Korea) in a microcentrifuge tube. After standing in a cold ice-bath for 30 min, whole homogenates were centrifuged at 13000 g for 5 min. The supernatant was collected, and the protein concentrations of whole homogenates were determined using the BCA Protein Assay kit (Pierce Biotechnology, Rockford, IL, USA).

#### 2.5.8. Isolation of Cytosolic and Nuclear Proteins

NE-PER (Pierce Biotechnology) was used for extraction of nuclear and cytosolic fractions according to the manufacturer's instructions. Briefly, isolated stomach tissue was homogenized in cold Cytoplasmic Extraction Reagent (CER)1 (with protease inhibitor cocktail set III; Calbiochem, La Jolla, CA, USA). After incubation for 10 min, CER2 was added to break down the cytoplasmic membrane. After centrifuging (16000 g for 5 min), the cytoplasmic extract was collected. Nuclear Extract Reagent with protease inhibitor cocktail set III (Calbiochem) was added to the remaining nuclear pellet and, after a 40 min incubation and centrifugation (16000 g for 10 min), the nuclear extract was harvested. Protein concentrations were determined using the BCA Protein Assay kit (Pierce Biotechnology).

#### 2.5.9. Immunoblots

Protein samples were loaded on 10–15% polyacrylamide gels and were then separated by SDS/PAGE and transferred to polyvinylidene fluoride membranes (Millipore, Billerica, MA, USA) using the Semi-Dry Trans-Blot Cell (Biorad Laboratories, Hercules, CA, USA). After transfer, the membranes were washed with 0.1% Tween-20 in 1× Tris-buffered saline (TBS/T) and blocked for 1 h at room temperature with 5% (w/v) skim milk powder in TBS/T. The blots were then incubated overnight at 4°C with primary antibodies. After washing three times for 5 min each in TBS/T, the membranes were incubated with appropriate secondary antibodies for 1 h at room temperature and detected using an ECL detection system (iNtRON Biotechnology Inc.), according to the manufacturer's instructions. ImageQuant TL software (Amersham Biosciences/GE Healthcare, Piscataway, NJ, USA) was used for densitometric evaluation of visualized immunoreactive bands. Primary antibodies against NF-*κ*B/p65 (1 : 1000 dilution, Santa Cruz Biotechnology, Santa Cruz, CA, USA) and I*κ*B-*α* (1 : 1000 dilution, Santa Cruz Biotechnology) were used and the signals were standardized to *β*-actin (1 : 2000 dilution, Sigma Chemical Co.) for cytosolic fraction and lamin B1 (1 : 2500 dilution, Abcam, Cambridge, UK) for nuclear fraction.

#### 2.5.10. RNA Extraction and Reverse Transcription Polymerase Chain Reaction

Total RNA was extracted and first strand cDNA was synthesized by reverse transcription (RT) using oligo (dT) primers and SuperScript II RNase H-Reverse Transcriptase (Invitrogen Tech-Line, Carlsbad, CA, USA). PCR reactions were in 20 *μ*L volumes with diluted cDNA sample. The final reaction concentration of sense and antisense primers was 10 pM; dNTP mix of 250 *μ*M; 10 × PCR buffer; and Ex Taq DNA polymerase of 0.5 U/reaction. Polymerase chain reaction (PCR) was performed with an initial denaturation step at 94°C for 5 min and a final extension step at 72°C for 7 min in the GeneAmp 2700 thermocycler (Applied Biosystems, Foster City, CA, USA). The primers used for cDNA amplification were 5′-AGCCCACGTCGTAGCAAACCACCAA-3′ (sense) and 5′-ACACCCATTCCCTTCACA GAGCAAT-3′ (antisense) for TNF-*α*; 5′-GAA AGT CAA CTC CAT CTG CC-3′ (sense) and 5′- CAT AGC ACA CTA CGT TTG CC-3′ (antisense) for IL-6; 5′-TCT CAC GTC ACT GA C TGT A-3′ (sense) and 5′-CTT TCA GTG TTG TGA GCG T-3′ (antisense) for IL-4; 5′-TGT GGG TCT GTT GTA GGG-3′ (sense) and 5′-GTG AGG TAA GAT GGT GGC-3′ (antisense) for IL-8. Amplification cycling conditions were as follows: 32 cycles at 94°C (30 s), 56°C (30 s), and 72°C (60 s) for TNF-*α*; 32 cycles at 94°C (30 s), 65°C (45 s), and 73°C (60 s) for IL-6; 40 cycles at 94°C (30 s), 54°C (30 s), and 72°C (30 s) for IL-4, IL-8, and *β*-actin. Following RT-PCR, 10 *μ*L samples of the PCR products were visualized by ultraviolet illumination after electrophoresis through 1.5% agarose gel and ethidium bromide staining. SLP Mylmager (UVP Inc., Upland, CA, USA) and ImageQuant TL (Amersham Biosciences/GE Healthcare) were used for semiquantitative analysis of the intensity of each PCR product.

### 2.6. Statistical Analysis

The overall significance of results was examined using one-way analysis of variance (ANOVA). Differences between groups were considered statistically significant at *P* < 0.05 with the appropriate Bonferroni correction made for multiple comparisons. All results are presented as means ± SEM.

## 3. Results

### 3.1. Antiulcerogenic Activities

#### 3.1.1. HCl/EtOH-Induced Gastric Ulcer

Intragastric administration of acidified EtOH resulted in multiple hemorrhagic lesions in the glandular portion of the stomach, with a lesion index of 81.8 ± 12.0 mm^2^. Treatment with GC-7101 at 25, 50, or 100 mg/kg resulted in a significant reduction in ulcer lesions, with 50.5%, 60.0%, and 48.8% ulcer inhibition, respectively. Animals that received 30 mg/kg of ranitidine or rebamipide also showed significant reductions in ulcer lesions, with 50.7% and 57.2% ulcer inhibition (Table 1).

#### 3.1.2. Indomethacin-Induced Gastric Ulcer

Rats receiving indomethacin had linear hemorrhagic lesions along the long axis of the stomach, with a lesion index of 35.6 ± 5.3 mm. Treatment with GC-7101 at 25, 50, and 100 mg/kg significantly attenuated indomethacin-induced ulcer lesions by 63.3%, 66.4%, and 72.9%. Administration of 30 mg/kg of ranitidine and rebamipide also provided significant protection, with 89.8% ulcer inhibition for ranitidine and 66.4% for rebamipide (Table 1).

#### 3.1.3. WIRS-Induced Gastric Ulcer

WIRS induced linear and circular ulcers in the glandular portion of stomach with coagulated blood at the bases, with a lesion index of 21.1 ± 2.1 mm. Treatment with GC-7101 at 25, 50, and 100 mg/kg resulted in marked decreases in gastric lesions, with 48.3%, 60.2%, and 60.6% ulcer inhibition. Animals that received 30 mg/kg of ranitidine and rebamipide were also significantly protected against WIRS-induced gastric lesions with 64.4% ulcer inhibition for ranitidine and 36.6% for rebamipide (Table 1).

#### 3.1.4. Acetic-Acid-Induced Subchronic Gastric Ulcer

Injection of acetic acid into the submucosal layer of the stomach induced round ulcer lesions at eight days after induction, with a lesion index of 20.0 ± 1.5 mm^2^. Treatment with GC-7101 at 25, 50, and 100 mg/kg for eight consecutive days significantly decreased the ulcer index with 55.6%, 57.6%, and 73.7% ulcer inhibition. Animals that received 30 mg/kg of either ranitidine or rebamipide also showed a significant reduction in ulcer lesions (48.4% ulcer inhibition for ranitidine and 57.7% for rebamipide) (Table 1).

### 3.2. Gastric Wall Mucus Secretion

**Figure 1:**
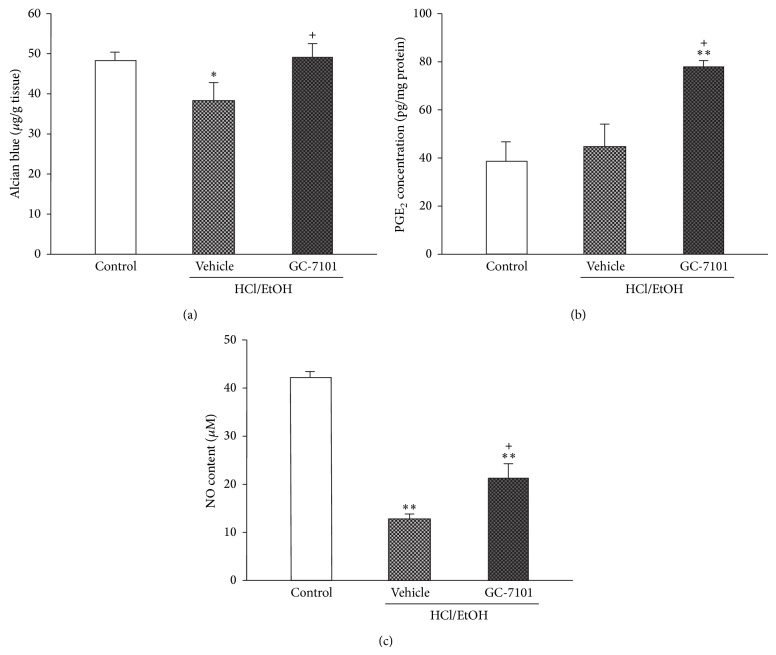
Effects of GC-7101 on gastric wall mucus content (a), mucosal PGE_2_ concentration (b), and mucosal NO content (c) in HCl/EtOH-induced gastric ulcer. The values are represented as means ± SEM for eight rats per group. ^∗,∗∗^Significantly different (*P* < 0.05, *P* < 0.01) from vehicle-treated control group. ^+^Significantly different (*P* < 0.05) from vehicle-treated HCl/EtOH group.

Rats treated with HCl/EtOH showed a significant decrease in the Alcian blue binding capacity of the gastric wall mucus (38.4 ± 4.4 *μ*g/g tissue) compared to vehicle-treated control rats (48.3 ± 2.1 *μ*g/g tissue). Treatment with 50 mg/kg of GC-7101 significantly increased the Alcian blue binding capacity of the gastric wall mucus to 127.9% of the vehicle-treated HCl/EtOH group (Figure 1(a)).

### 3.3. Mucosal PGE_2_ Level in HCl/EtOH-Treated Rats

In vehicle-treated control rats, the mucosal PGE_2_ level remained at basal level (38.3 ± 8.5 pg/mg protein). Treatment with HCl/EtOH showed a tendency toward increasing mucosal PGE_2_ levels to 117.9% of the basal level. Treatment with GC-7101 augmented this increase to 172.3% of the vehicle-treated HCl/EtOH group (Figure 1(b)).

### 3.4. Mucosal NO Level in HCl/EtOH-Treated Rats

In vehicle-treated control rats, the mucosal NO level was at basal levels (42.1 ± 0.1 *μ*M). Treatment with acidified EtOH dramatically reduced the mucosal NO level to 30.7% of the basal level. However, treatment with 50 mg/kg of GC-7101 significantly attenuated this reduction to 164.7% of the vehicle-treated HCl/EtOH group (Figure 1(c)).

### 3.5. Anti-Inflammatory Properties

#### 3.5.1. MPO Activity in HCl/EtOH-Treated Rats

**Figure 2:**
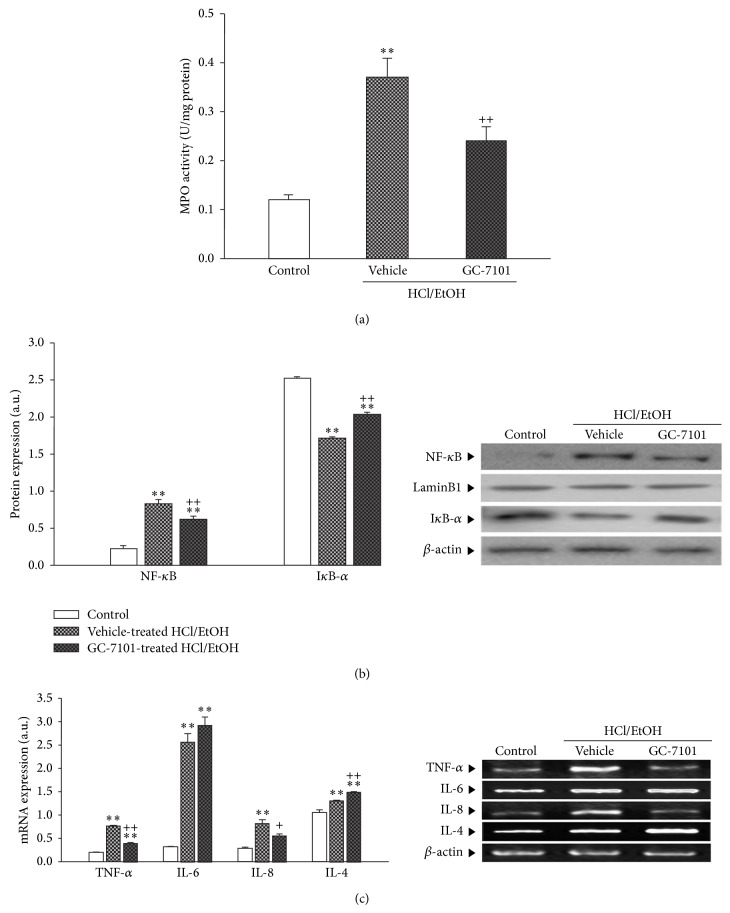
Effects of GC-7101 on MPO activity (a), nuclear translocation of NF-*κ*B (b), and inflammatory cytokines mRNA expression (c) in HCl/EtOH-induced gastric ulcer. Immunoblot and PCR shown are representatives of at least three experiments with similar results. ^**^Significantly different (*P* < 0.01) from vehicle-treated control group. ^+,++^Significantly different (*P* < 0.05, *P* < 0.01) from vehicle-treated HCl/EtOH group.

HCl/EtOH treatment resulted in a significant increase in MPO activity to 308.3% of the vehicle-treated control group. The 50 mg/kg GC-7101-treated group showed a marked attenuation in this increase to 64.9% of the vehicle-treated HCl/EtOH group (Figure 2(a)).

#### 3.5.2. Nuclear NF-*κ*B and Cytosolic I*κ*B-*α* Protein Expression in HCl/EtOH-Treated Rats

As shown in Figure 2(b), the nuclear translocation of NF-*κ*B in vehicle-treated control rats is quite low. HCl/EtOH treatment resulted in a significant increase in nuclear translocation of NF-*κ*B to 379.6% of the vehicle-treated control group. The 50 mg/kg GC-7101-treated group showed marked attenuation in this increase to 75.4% of the vehicle-treated HCl/EtOH group. In contrast, the HCl/EtOH-treated group showed a dramatic reduction in cytosolic I*κ*B-*α* protein expression by 67.7% of the basal level. GC-7101 treatment markedly attenuated this reduction to 119.0% of the vehicle-treated HCl/EtOH group.

#### 3.5.3. TNF-*α*, IL-6, IL-8, and IL-4 mRNA Expression in HCl/EtOH-Treated Rats

Rats receiving HCl/EtOH showed significant increases in inflammatory mediator mRNA expression compared to the vehicle-treated control group: 375.6% of the vehicle-treated control group for TNF-*α*, 823.0% for IL-6, 288.8% for IL-8, and 124.1% for IL-4. Although 50 mg/kg of GC-7101 did not affect the increase in IL-6 mRNA, it markedly attenuated TNF-*α* level to 52.3% of the vehicle-treated HCl/EtOH group and IL-8 to 68.4% of the vehicle-treated HCl/EtOH group. However, treatment with GC-7101 significantly augmented IL-4 mRNA to 114.1% of the vehicle-treated HCl/EtOH group (Figure 2(c)).

### 3.6. Antioxidant Activities

#### 3.6.1. Lipid Peroxidation and Glutathione Content in HCl/EtOH-Treated Rats

In vehicle-treated control rats, MDA in stomach tissue was at basal levels (0.4 ± 0.1 nmol/mg protein). HCl/EtOH treatment resulted in markedly increased MDA levels to 350% of the basal levels. Treatment with 50 mg/kg GC-7101 significantly attenuated this increase by 57%. The GSH/GSSG ratio in stomach tissue isolated from vehicle-treated control rats was at basal levels (18.7 ± 1.0), and EtOH/HCl treatment significantly reduced the GSH/GSSG ratio by 64.2% of the basal level. Treatment with 50 mg/kg of GC-7101 completely restored reduced GSH/GSSG ratio to 175.8% of the vehicle-treated HCl/EtOH group (Table 2).

#### 3.6.2. SOD and CAT Activities in HCl/EtOH-Treated Rats

In vehicle-treated control rats, SOD activity remained at basal levels (97.0 ± 0.1 U/g protein). The HCl/EtOH-treated group showed a significant reduction in SOD activity to 67.6% of the basal level. This was attenuated by treatment with 50 mg/kg of GC-7101 (155.5% of the vehicle-treated HCl/EtOH group). Similarly, CAT activity in vehicle-treated control rats was at basal levels (22.0 ± 2.1 *μ*mol/min/mg protein) and HCl/EtOH treatment resulted in a dramatic reduction in CAT activity to 54.1% of the basal level. However, treatment with 50 mg/kg of GC-7101 significantly restored this decrease to 158.8% of the vehicle-treated HCl/EtOH group (Table 2).

## 4. Discussion

This study investigated the antiulcerogenic effect of GC-7101, an ethyl acetate fraction of* Flos Lonicerae*, against* in vivo* acute and subchronic gastric ulcers. We demonstrated the molecular mechanisms of its antiulcerogenic action: enhanced cytoprotection accomplished by induction of gastric mucin secretion and PGE_2_ biosynthesis and antioxidant and anti-inflammatory properties.

Major etiologic factors of peptic ulcers include irregular eating habits, alcohol consumption, excessive use of drugs such as nonsteroidal anti-inflammatory drugs, psychological and/or physiological stress, and* Helicobacter pylori *infection [21]. Since ethanol, indomethacin, and stress are all representative causative factors of human gastric ulcers, we evaluated the effect of GC-7101 on experimental models of acute gastric ulcer induced by EtOH/HCl, indomethacin, and WIRS. GC-7101 markedly reduced gastric lesions induced by EtOH/HCl, indomethacin, and WIRS at all doses administered, demonstrating significant gastroprotection against acute gastric ulcers.

Human gastric ulcers are characterized by repeated recurrence or relapse and this is largely associated with the quality of healing of the preceding ulcer. Acetic-acid-induced gastric ulcers in rats closely resemble chronic ulcers in humans, particularly in the healing process [22]. Ulcer healing is a dynamic process of filling the mucosal defect with epithelial and connective tissue cells, for which complex biological responses including cell proliferation, migration, regeneration, active angiogenesis, and extracellular matrix deposition are required [23]. In the present study, GC-7101 significantly reduced the area of the acetic-acid-induced gastric injury. Interestingly, 100 mg/kg GC-7101 showed an even higher ulcer inhibition rate than rebamipide (positive control), suggesting the strong ulcer healing effect of GC-7101.

Administration of EtOH, a necrotizing agent, not only causes direct insult to the stomach but also disrupts the balance in various factors of the gastric mucosa by increasing ROS formation and decreasing gastric mucus and prostaglandin production and gastric mucosal blood flow [24]. The mucus has a structural role in creating an undisturbed layer on the mucosal surface that supports maintenance of a near-neutral pH at the surface and acts as a physical barrier against luminal pepsin [25]. Previous reports suggested that mucus retards the diffusion of protons, which would aid in maintaining a favorable pH at the apical surface of the epithelium [26]. According to our unpublished data, administration of GC-7101 did not affect basal or stimulated acid secretion at a fully cytoprotective dose. However, in the present study, EtOH/HCl significantly decreased gastric mucus/mucin secretion, and GC-7101 attenuated this reduction, indicating an enhancement of the mucosal defense system by GC-7101.

Emerging evidences support the notion that the enhancement in PGE_2_ and NO levels is a protective mechanism against gastric mucosal damage [27, 28]. PGE_2_ biosynthesis in the gastrointestinal tract is exclusively catalyzed by constitutive form of cyclooxygenase- (COX-) 1, whereas COX-2 mainly yields PGs in pathophysiological reactions such as inflammation [29]. Disrupted synthesis of the PGE_2_ by inhibition of COX-1 in the mucus is considered one of the main mechanisms of the formation of peptic ulcers and duodenal ulcers [30]. On the other hand, COX-2 is highly expressed in the majority of gastric mucosa-associated lymphoid tissue [31] and its inhibition was suggested to be beneficial [32]. NO is a mediator that plays a crucial role in regulating the defense of the gastric mucosa; NO is involved in the mucus secretion [33], inhibiting neutrophil aggregation [34] and enhancing mucus flow [35]. Recently, it was shown that augmentation of the eNOS/iNOS ratio is one of the key mechanisms in ulcer healing from indomethacin-induced gastritis [36]. Thus, the sources and role of PGE_2_ and NO are highly complicated in the pathological conditions due to the involvement of various enzymes in their synthetic processes. In our study, rats treated with EtOH/HCl showed a tendency to increase mucosal PGE_2_ levels while other studies reported decreased mucosal PGE_2_ levels after treatment with necrotizing agents [2]. This discrepancy might be because the mucosal PG content depends on the severity of the insult. 40% EtOH increases PG levels while 100% EtOH inhibits mucosal PGE_2_ [37]. Indeed, when we increased the amount to 1.5 mL of 150 mM HCl in 60% EtOH in our preliminary studies, the mucosal PGE_2_ level was significantly reduced compared to that in vehicle-treated control rats (data not shown). GC-7101 treatment markedly augmented the mucosal PGE_2_ level in this study. Furthermore, treatment of EtOH/HCl markedly decreased the mucosal NO content, and this was significantly attenuated by GC-7101. These results suggest that the gastroprotection by GC-7101 could involve general cytoprotection from increased mucosal PGE_2_ and consistent mucosal NO content. Interestingly, we recently reported that chlorogenic acid, a bioactive component of GC-7101, significantly inhibited iNOS and COX-2 protein levels in surgically induced reflux esophagitis [38].

Gastric ulcers are considered to be the manifestation of an inflammatory response. Gastric inflammation increases leukocyte adherence to the endothelial surface of postcapillary venules and is characterized by the migration of macrophages and polymorphonuclear leukocytes in the ulcer area. Migrated macrophages release proinflammatory mediators such as TNF-*α* and IL-6, presumably by transcriptional regulation through NF-*κ*B, and this upregulates leukocyte recruitment and transmigration in gastric inflamed areas [39]. NF-*κ*B activation and subsequent proinflammatory cytokine production, particularly TNF-*α* and IL-8, are evident in patients with gastric ulcer disease, especially those positive for* H. pylori* [8]. In our recent study, ethanol extract of* Flos Lonicerae* significantly reduced NF-*κ*B activation, downstream of toll-like receptor 4, in the liver and lung of septic mice [40]. However, IL-4 downregulates a wide variety of TNF-induced effects, including suppression of TNF-induced NF-*κ*B and AP-1 activation, and inhibition of apoptotic caspase-3 activity [41]. In this context, the ulcer healing effect of sucralfate is partially the result of rapid mucosal IL-4 generation that leads to suppression of mucosal apoptotic events [42]. Our results indicate that the administration of acidified EtOH resulted in a dramatic increase in NF-*κ*B translocation accompanied by an increase in proinflammatory cytokines. GC-7101 significantly attenuated the nuclear translocation of NF-*κ*B and further decreased TNF-*α* and IL-8 mRNA expression. Rats treated with acidified EtOH showed an increase in IL-4 mRNA expression that was augmented by GC-7101. These results indicate that anti-inflammatory properties of GC-7101 might contribute to its antiulcerogenic activity against EtOH/HCl-induced ulcers.

Oxidative stress is responsible for the loss of mucosal integrity caused by various aggressive factors including EtOH [43]. The gastric mucosa contains a relatively high concentration of GSH and is considered a first-line nonenzymatic defensive mechanism against oxidative stress in the gastric mucosa. Previous reports demonstrated that sulfhydryl blockers reverse cytoprotection by PGF_2*β*_ against absolute EtOH-induced gastric lesions, suggesting that gastric sulfhydryl compounds, primarily GSH, are essential in gastric cytoprotection [44]. In addition, the enzymatic antioxidant defense system of SOD and CAT scavenges and regulates overall ROS to maintain physiological homeostasis. When SOD is inhibited with diethyl thiocarbamate, bicarbonate secretion is reduced and gastric lesion formation is increased [45]. SOD and CAT levels are significantly reduced in patients with gastric and duodenal ulcers and even in gastric cancer patients [46]. Previously,* Flos Lonicerae* extract and chlorogenic acid showed comparable effects of ROS reduction in perfluorooctane-sulfonate-treated human umbilical vein endothelial cells [47] and other bioactive components such as luteolin and caffeic acid demonstrated strong 1,1-diphenyl-2-picrylhydrazyl (DPPH) radical scavenging activity [13]. In our study, the stomach of rats treated with acidified EtOH showed a significant increase in MDA level, accompanied by a decrease in GSH/GSSG ratio and SOD and CAT activities. GC-7101 not only reduced stomach tissue lipid peroxidation but also increased endogenous antioxidants including the GSH/GSSG ratio and SOD and CAT activities, indicating that its antiulcerogenic properties are partially based on its antioxidant function.

It is well documented that one of the sources of ROS in gastric mucosal injury is the activated neutrophils; MPO is a heme enzyme that uses the oxidizing potential of superoxide and hydrogen peroxide to convert chloride ion to hypochlorous acid and other ROS from neutrophils [48]. Thus, MPO could be a key element responsible for oxidative damage in the gastric mucosal inflammation. Previously,* Flos Lonicerae* was also shown to suppress inflammatory cells infiltrations in reflux esophagitis-induced injured tissue [10]. In the present study, neutrophil infiltration in stomach tissue significantly increased as evidenced by MPO activity and this increase was attenuated by GC-7101.

## 5. Conclusions

In conclusion, we demonstrated the antiulcerogenic effect of GC-7101, an ethyl acetate extract of* Flos Lonicerae*, against various experimental ulcer models. This effect seemed to involve enhancement of gastric defensive mechanisms against aggressive factors, as evidenced by increased mucin secretion, mucosal PGE_2_ biosynthesis, and NO. Furthermore, the nonspecific cytoprotection afforded by GC-7101 coincided with enhancement of endogenous antioxidative mechanisms and marked anti-inflammatory properties. The overall data in conjunction with the absence of acute toxicity support GC-7101 as a potential therapeutic medication for the care of clinical gastric ulcer patients.

## Figures and Tables

**Table 1:** Antiulcerogenic activities of GC-7101 for acute and subchronic gastric ulcers.

Experimental models	Treatment	Dose	Gastric lesion	Inhibition (%)
HCl/EtOH	Vehicle	—	81.8 ± 12.0	—
	Ranitidine	30	40.3 ± 7.9^**^	50.7
	Rebamipide	30	35.0 ± 8.3^**^	57.2
	GC-7101	25	40.5 ± 6.8^**^	50.5
		50	32.8 ± 4.4^**^	60.0
		100	41.9 ± 8.4^*^	48.8

Indomethacin	Vehicle	—	35.6 ± 5.3	—
	Ranitidine	30	3.6 ± 0.9^**^	89.8
	Rebamipide	30	12.0 ± 2.1^**^	66.4
	GC-7101	25	13.1 ± 2.1^**^	63.3
		50	12.0 ± 2.1^**^	66.4
		100	9.6 ± 4.9^**^	72.9

Water immersion restraint stress	Vehicle	—	21.1 ± 2.1	—
	Ranitidine	30	7.5 ± 1.1^**^	64.4
	Rebamipide	30	13.4 ± 1.5^*^	36.6
	GC-7101	25	10.9 ± 1.4^**^	48.3
		50	8.4 ± 1.1^**^	60.2
		100	8.3 ± 1.5^**^	60.6

Acetic acid	Vehicle	—	20.0 ± 1.5	—
	Ranitidine	30	10.3 ± 1.1^**^	48.4
	Rebamipide	30	8.4 ± 1.5^**^	57.7
	GC-7101	25	8.9 ± 1.7^**^	55.6
		50	8.5 ± 1.9^**^	57.6
		100	5.3 ± 0.8^**^	73.7

The values are represented as means ± SEM for eight to ten rats per group. ^∗,∗∗^Significantly different (*P* < 0.05, *P* < 0.01) from vehicle-treated rats.

**Table 2:** Antioxidant properties of GC-7101 for HCl/EtOH-induced gastric ulcers.

Group	MDA (nmol/mg protein)	GSH/GSSG ratio	SOD (U/g protein)	Catalase (*μ*mol/min/mg protein)
Vehicle-treated control	0.4 ± 0.1	18.7 ± 1.0	97.0 ± 0.1	22.0 ± 2.1
HCl/EtOH				
Vehicle	1.4 ± 0.2^**^	12.0 ± 0.7^*^	65.6 ± 0.3^*^	11.9 ± 1.3^**^
GC-7101 50 mg/kg	0.8 ±0.2^+^	21.1 ± 3.1^++^	102.0 ± 12.8^+^	18.9 ± 2.1^+^

The values are represented as means ± SEM for eight rats per group. ^∗,∗∗^Significantly different (*P* < 0.05, *P* < 0.01) from vehicle-treated control group. ^+,++^Significantly different (*P* < 0.05, *P* < 0.01) from vehicle-treated HCl/EtOH group.
